# The immunopeptidome of colon cancer cells treated with topoisomerase inhibiting drug reveals differential as well as common endogenous protein sampling and display of MHC I-associated peptides

**DOI:** 10.1080/23723556.2025.2471640

**Published:** 2025-03-03

**Authors:** Deepa Bedi, Mohammed Hassan, Alehegne Yirsaw, Biba Vikas, Pran Datta, Temesgen Samuel

**Affiliations:** a Departments of Pathobiology and Biomedical Sciences, Tuskegee University, College of Veterinary Medicine and Center for Biomedical Research, Tuskegee, AL, USA; b University of Alabama at Birmingham, Birmingham, AL, USA

**Keywords:** MHC I, immunopeptidome, colorectal cancer, topoisomerase inhibitor, microsatellite

## Abstract

Immunotherapy options for microsatellite stable (MSS) colorectal cancer are currently very limited. The lack of detectably unique or altered immunogens in the tumor microenvironment may be a factor. Radiation and chemotherapy may enhance immunotherapy by increasing cancer cell visibility through Major Histocompatibility Complex I (MHC I) expression. To investigate this, we treated MSS and microsatellite-instable (MSI) colon cancer cells with a topoisomerase inhibitor and analyzed MHC I-associated peptides. Treatment increased peptide numbers by 5% in RKO (MSI) cells and 83% in SW620 (MSS) cells, with 40–50% of peptides being exclusive to treatment. Additionally, clustering analysis revealed a set of peptides with uniquely conserved residues displayed only in treated MSS SW620 cells. Gene Ontology analysis of MHC I-displayed proteins revealed a treatment-induced increase in extracellular vesicle- and nuclear-derived proteins, alongside reduced cytosolic protein sampling. Overall, we present evidence for treatment-inducible differential display of peptides, some of which may affect interactions and functions of immune cells. Given the multitude of factors that modulate the effects of increased MHC I expression and associated peptides, further studies are needed to elucidate the pathophysiological implications of these changes.

## Introduction

Colorectal cancer (CRC) is one of the types of cancers for which immunotherapy options are very limited. Only a very small proportion of patients, mainly microsatellite instable (MSI) subtypes, benefit from the currently available immunotherapies that inhibit the immune checkpoint mechanism.^1–3^ Microsatellite stable (MSS) tumors have very limited or no such options. The reasons for the ineffectiveness and disparity are not fully understood, but common hypotheses include the lack of cancer cell neo-epitopes that T-cells could recognize, and the existence of heavy immunosuppression in the tumor microenvironment.^4–8^ Therefore, to date chemotherapy remains the basis for standard of care for advanced cases that are not managed by surgery.

T-cells are the key players for anti-tumor immunity. Cytotoxic T-cells distinguish between self and foreign or altered self-cells by their ability to recognize peptides displayed on MHC I molecules present on all nucleated cells in the body. When a cytotoxic T-cell receptor binds to an MHC I-peptide complex displayed on a cancer cell, the T-cell will be activated, will proliferate, and attack the target cell.^9–12^ Since almost all self-reactive T-cells are eliminated in the thymus during selection and development, healthy individuals or tissues are deficient in auto-reactive cytotoxic T-cells.^13–15^

Since tumor cells originate from altered cells in the body, the immune system faces challenges to recognize and eliminate cancerous cells. Besides, both tumor cells and other cells in solid-tumor microenvironment (TME) often create an ambience where T-cells are immunosuppressed or excluded, creating additional challenges.^7,16,17^ Therefore, multiple strategies are being developed to address these and other challenges that limit the effectiveness of immunotherapy for solid tumors.

The ultimate goal of immunotherapy is to enable targeting of cancerous cells by patient’s own immune cells. Studies have shown that radiation and chemotherapy can render cancer cells appear ‘foreign’ to cytotoxic T-cells, and hence visible for immune attack.^18–22^ Ideally, the therapy must sufficiently disrupt the translated genome and alter the cancer cells so that neo-epitopes (antigenic peptides) are generated from endogenous proteins. While this outcome is possible, it may come at a cost of side effects and toxicity if the treatment is not selective enough to spare normal cells.^23–25^ While this challenge remains common to many cancer therapies, selective and targeted delivery strategies are also evolving. Another approach is to determine the optimal combination therapy timing and dosage range that is effectively lethal to cancer cells, or to prime effector immune cells with drugs or vaccines.^26–33^

In this study, to understand how chemotherapy alters the diversity of endogenous peptides displayed in association with MHC I, we examined peptides eluted from MHC I from control or CPT-treated SW620 (MSS) and RKO (MSI) colon cancer cells. We present data on the most relevant differentially or commonly displayed peptides and proteins, as well as cellular components sampled for peptide display.

## Materials and methods

### Cell lines and culture

SW620 (Microsatellite Stable, MSS), and RKO (Microsatellite Instable, MSI) cells of colorectal cancer origin were used in this study. The cells were purchased from the American Type Cell Culture (ATCC) collection. Multiple aliquots of early passages from the original vials were stored frozen in liquid nitrogen and retrieved for experiments. No additional validations were done. Both cell lines were grown in Dulbecco’s Modified Eagle’s Medium (DMEM) culture medium from Corning (Corning, NY, USA) containing 10% fetal bovine serum plus 10 µg/ml ciprofloxacin (MilliporeSigma, St Louis, MO, USA).

### Treatment and harvesting and storage

For this study, cells were treated in multiple T25 culture flasks with the topoisomerase inhibitor drug camptothecin (CPT) at 0.5 µM concentration or similar volume of the carrier dimethyl sulfoxide (DMSO, MilliporeSigma, USA) for 24 h.

After the treatment, the culture medium was removed by aspiration, and cells were harvested by brief trypsinization followed by neutralization with complete medium containing FBS. The cells were collected in a 15 ml tube centrifuged for 2 min at 3000 rpm (about 750*g*). After one round of wash, the final cell count was adjusted, by using an automated cell counter, to 100 million cells for each of the control or treated cells, and centrifuged again in 2 mL Eppendorf tubes. The final cell pellets were snap frozen and stored. Snap freezing was carried out in a CoolRack module pre-cooled to −20°C for approximately 5–7 min. The module was then transferred to a base containing liquid nitrogen and equilibrated for approximately 12–15 min. The samples in the 2 mL tubes were placed in the CoolRack module for 3–4 min until frozen. Frozen samples were stored at −80°C until shipped on dry ice for bioanalysis (Cayman Chemicals, Ann Arbor MI, USA).

### Immunopeptidome analysis

Bioanalysis for the identification of peptides associated with MHC I was conducted at Cayman Chemicals. After receipt of the control and treated cells, the process included cell lysis, immunoaffinity chromatography specific to human MHC class I, elution of bound peptides, and identification of the eluted peptides by mass spectrometry. The experimental protocol by Cayman Chemicals is provided as a Supplementary Material. Peptides were detected at the 1% peptide-to-spectrum matches (PSM) false discovery rate (FDR), based on forward/decoy database searching. A data file containing the full list of peptides, their peak intensity, and other features is available upon request. Additionally, the peptide list from each sample was submitted to GibbsCluster tool (https://services.healthtech.dtu.dk/service.php?GibbsCluster-2.0) for motif analysis.

### Gene ontology and enrichment analysis

The accession numbers of parental proteins from which the peptides were derived were used to build a separate nonredundant list of proteins. These parental proteins were considered to be sampled and processed for display of their peptides in association with MHC I at the cell surface. The g:GOSt functional profiling tool of g:Profiler (https://biit.cs.ut.ee/gprofiler/gost) was used to perform functional enrichment (over-representation) analysis of proteins on the list. Cellular Component (CC) database was selected to profile the proteins according to their components of origin. Only the top 20 CC categories among the significant (-Log10adj *p* > 2.0) were included for graphic display in the results section.

### Protein modeling of S10A9

The S10A9 (S100A9) model was re-created in SWISS-MODEL (https://swissmodel.expasy.org/interactive) to interactively select a range of amino acid residues of interest for display. An existing structure of the same protein (also named Calprotectin, PDB ID 6ds2.1.B) was used as a template for this purpose. A range of residues comprising peptides identified in this study were colored red or blue.

## Results

Quantitative and qualitative analyses were performed to enumerate, identify the source proteins, and the subcellular localization and functions of the identified peptides.

### CPT treatment increases the overall number of unique peptides displayed on colon cancer cells

**Figure 1.**
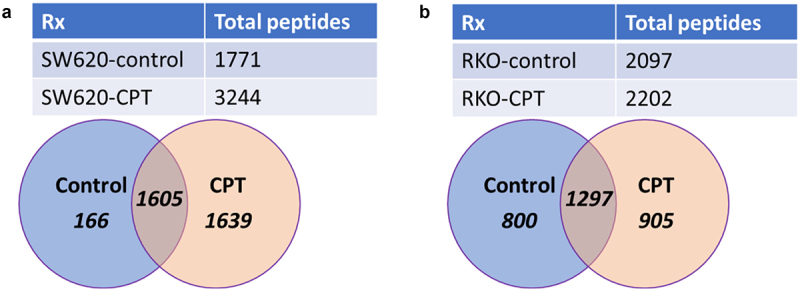
Total and unique peptides displayed on MHC I in control or cpt-treated SW620 (a) or RKO (b) colon cancer cell lines. SW620 and RKO cells were treated with CPT or vehicle (control) and peptides displayed on the MHC I molecules were analyzed. The number of total, unique to control or CPT, or common peptides between the two treatment groups are shown.

Enumeration of the peptides eluted from the MHC I molecules on SW620 and RKO cells showed that the total number of peptides displayed on treated cells increased by only 5% in RKO cells compared to 83% increase in SW620 cells (Figure 1a-b). Similarly, about 50% of the peptides displayed on CPT-treated SW620 cells and about 41% on CPT-treated RKO cells were exclusive to the treated cells, indicating a large pool of unique peptides associated with MHC I only after cells were treated with the drug. On the other hand, 43% and 47% of total unique peptides in RKO and SW620 cells, respectively, were displayed on MHC I molecules irrespective of the treatment conditions.

### Variable length and increased number of post translationally modified peptides presented on MHC I on treated cells

**Figure 2.**
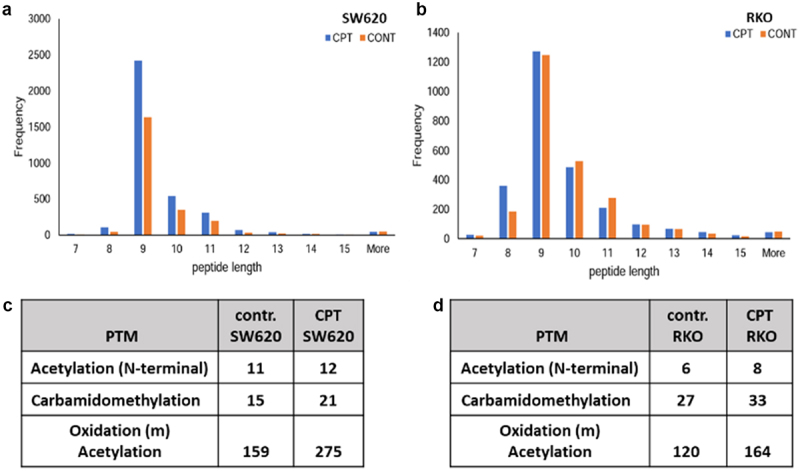
Length distributions (a-b) and post translational modification (PTM) profiles (c-d) of peptides displayed on MHC I in control or cpt-treated SW620 (a and c) or RKO (b and d) colon cancer cell lines.

MHC I molecules may bind to peptides of variable lengths, but because of the biochemical and biophysical natures of the peptide-binding grooves, 9-mer peptides preferentially bind and are, therefore, displayed on cell surface. As the distribution of peptide length shows (Figure 2a-b), as expected, 9-mers constitute the abundant majority of peptides displayed on both cell types. Among peptides of 8–11 amino acids length, CPT-treated SW620 cells consistently displayed a higher number of peptides compared to the control. On the other hand, CPT-treated RKO cells displayed more of 8-mer and 9-mer peptides but lesser of 10-mer and 11-mer peptides in comparison to the corresponding controls. Additionally, the overall display of post translationally modified (PTM) peptides was increased by about 34% (RKO) and 66% (SW620) after treatment with CPT (Figure 2c-d).

### A variety of peptides show treatement-restricted association with MHC I on one or both cell lines

Next, we queried if any of the treatment-restricted peptides displayed on MHC-I or the parental proteins from which the peptides were derived, were common between the SW620 and RKO cells. For this, we first sorted the peptides for their occurrence only in treated cells, and compared the protein length in the number of amino acids (AA), molecular size in kilodaltons (kDa), if the treatment-restriction was in one or both cell types, and subcellular localization. As shown in Table 1, we found nine peptides displayed by both cell types, although only two peptides, derived from LSG1 and TXNIP proteins, were treatment-restricted in both. The remaining seven were treatment-restricted only in RKO cells. Two of the nine restricted peptides were from Histone proteins.

**Table 1.** Peptides presented on both RKO and SW620 cells, including some restricted to RKO cells treated with CPT. Accession numbers, names, and some features of the parent proteins are shown. Some of the peptides may originate from any of the listed family of related proteins.

Rx-restricted peptides in one or both SW620 and RKO	Protein (Accession #)	AA (full length), Mol. Mass (kDa)	Rx-restricted in	Comment	Subcellular localization (Uniprot)
RRAPAGGSL	Q9H089|LSG1	658 (75.2)	both	Large subunit GTPase 1 homolog	Cytoplasm, endoplasmic reticulum, Cajal body (nucleus)
TRVKAVRIL	Q9H3M7|TXNIP	391 (43.7)	both	Thioredoxin-interacting protein	Cytoplasm
FRVPTANVS	P04406|G3P	335 (36)	RKO	Glyceraldehyde-3-phosphate dehydrogenase	Cytoplasm, perinuclear, nucleus, cytoskeleton
IQRTPKIQVYSRHPA	P61769|B2MG	119 (13.7)	RKO	Beta-2-microglobulin	Cell surface, secreted
VKLAKAGKNQGD	P19338|NUCL	710 (76.6)	RKO	Nucleolin	Nucleoplus, cytoplasm granules
DAVTYTEHAK	P62805|H4	103 (11.4)	RKO	Histone H4	Nucleus, Chromosome
TTPSYVAFTDTER	P0DMV8|HS71A	641 (70)	RKO	Heat shock 70 kDa protein P0DMV9|HS71B_:P11142|HSP7C_:P34931|HS71L_:P17066|HSP76_:P54652|HSP72_:P48741|HSP77	Cytoplasm, Nucleus
IYVDDGLISLQVK	P14618|KPYM	531 (57.9)	RKO	Pyruvate kinase PKM	Cytoplasm, Nucleus
EIQTAVR	O60814|H2B1K	126 (13.9)	RKO	Histone H2B type 1; Peptide may originate from other H2B proteins: P06899|H2B1J_:P57053|H2BFS_:Q99879|H2B1M_:Q93079|H2B1H_:Q5QNW6|H2B2F_:Q99877|H2B1N_:P62807|H2B1C_:P58876|H2B1D_:Q99880|H2B1L_:P33778|H2B1B_:P23527|H2B1O_:Q16778|H2B2E_:Q96A08|H2B1A	Nucleus, chromosome

Similarly, we sorted out peptides restricted to treated SW620 cells, yet displayed also in RKO cells irrespective of treatment status. Here, we identified 14 peptides originating from 12 different parent proteins (Table 2). Three of the peptides were derived from the same region on the protein dermicidin (DCD), only varying in their lengths of 12, 13, and 15 amino acids. Interestingly, the distribution of lengths of peptides cross-displayed on both SW620 and RKO cells did not appear to correlate with the total peptide length distribution. In the total pool, 9-mer peptides constituted 50–70% (2144/4299 for RKO, and 3464/5015 for SW620) of the peptides displayed in each cell type. On the other hand, as can be seen in both Tables 1 and 2, only 10 of the 23 (43%) peptides were 9-mers among those displayed on both cells and treatment-restricted in either of the two cell types.

**Table 2.** Peptides restricted to only CPT-treated SW620 cells but displayed on both RKO and SW620 cells. Accession numbers, names, and some features of the parent proteins are shown. Some of the peptides may originate from any of the listed family of related proteins.

Rx-restricted SW620 peptides displayed on both cells	Protein (Accession #)	Mol. Mass (kDa)	Rx-restricted in	Comment	Subcellular localization (Uniprot)
EIIDLVLDR	Q9BQE3|TBA1C	449 (49.9)	SW620	Tubulin alpha; P68363|TBA1B: Q71U36|TBA1A	Cytoskeleton
TRNDYVM(+15.99)MY	Q96QU8|XPO6	1125 (128.9)	SW620	Exportin-6; Oxidation (M) M7:Oxidation (M):40.00	Nucleus, cytoplasm
ESVGKGAVHDVKDVL	P81605|DCD	110 (11.3)	SW620	Dermcidin	Secreted, membrane
ESVGKGAVHDVKD	P81605|DCD	110 (11.3)	SW620	Dermcidin	Secreted, membrane
GVVDSEDIPLNLSR	Q12931|TRAP1	704 (80.1)	SW620	HSP75 heat shock protein 75 kDa, mitochondrial; TNFR-associated protein 1 (TRAP1)	Mitochondria
GVVDSEDLPLNISR	P08238|HS90B	724 (83.3)	SW620	Heat shock protein HSP 90-beta; P07900|HS90A_:Q58FF7|H90B3	Cytoplasm, cell membrane, nucleus
EENPIVLEF	Q9BWU0|NADAP	796 (88.8)	SW620	Kanadaptin	Nucleus, Cytoplasm
LLLPGELAK	O60814|H2B1K	126 (13.9)	SW620	Histone H2B type 1 ; Peptide may originate from other H2B proteins: P06899|H2B1J:P57053|H2BFS:Q99879|H2B1M:Q93079|H2B1H:Q5QNW6|H2B2F:Q99877|H2B1N:P62807|H2B1C:P58876|H2B1D:Q99880|H2B1L:P33778|H2B1B:P23527|H2B1O:Q16778|H2B2E:Q8N257|H2B3B:Q96A08|H2B1A:A0A2R8Y619|H2BE1	Nucleus, chromosome
AVIVLVENFYK	Q96FQ6|S10AG	103 (11.8)	SW620	Protein S100-A16	Nucleolus, cytoplasm
KRFGKAYNL	Q86XP3|DDX42	938 (103)	SW620	ATP-dependent RNA helicase DDX42	Cytoplasm, Cajal body
TRMRHVISY	O14949|QCR8	82 (9.9)	SW620	Cytochrome b-c1 complex subunit 8	Mitochondria
IRKPYIWEY	P07814|SYEP	1512 (170.6)	SW620	Bifunctional glutamate/proline–tRNA ligase; EPRS1	Cytoplasm, membrane
LHTEAQIQEEGTVVELTGR	P01023|A2MG	1474 (163.3)	SW620	Alpha-2-macroglobulin	Secreted
GAVHDVKDVLDS	P81605|DCD	110 (11.3)	SW620	Dermcidin	Secreted, membrane

### Amino acid position conservation analysis of peptides associated with MHC I shows neo-peptide pattern in treated SW620 cells

**Figure 3.**
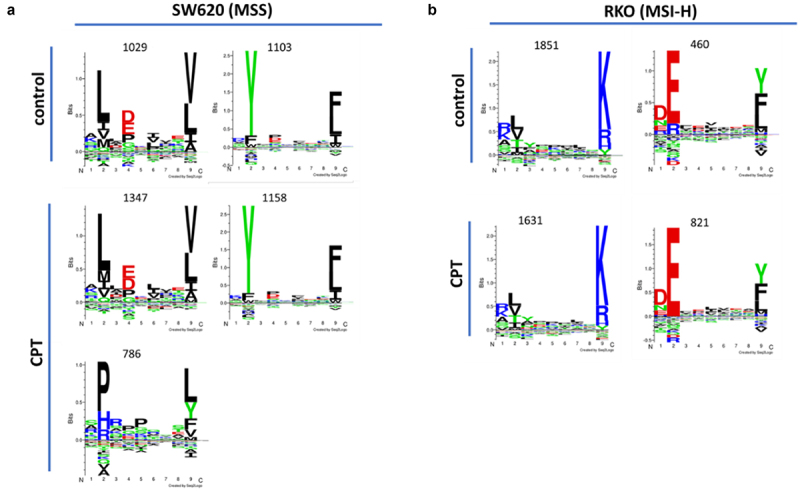
Conservation patterns of MHC I-binding 8-13-aa peptides in control or CPT-treated SW620 (A) or RKO (B) cells. Peptides eluted from MHC molecules in control or treated cells were analyzed by using Gibbs motif cluster analysis tool for positional conservation. CPT-treated SW620 cells display 786 peptides with unique motifs.

MHC I molecules are loaded primarily with peptides generated from endogenous proteins. Among peptides displayed along the MHC I groove, key amino acid positions needed for proper fitting

and binding of the peptides are relatively conserved for a given MHC I allele. Overall, amino acids at positions 2 and 9 on the peptide play dominant physical and biochemical roles in the peptide-MHC association. Therefore, we next analyzed the peptide repertoire eluted from the MHC I molecules in SW620 and RKO cells for their amino acid distributions, using the Gibbs motif cluster analysis. As shown in Figure 3. a-b, both control and CPT-treated SW620 or RKO cells maintained, irrespective of the treatment, a very conserved distribution of peptide motifs, including amino acids at the key positions. The exception to this was the occurrence of a separate cluster in SW620 cells treated with CPT. This cluster comprising a smaller pool of 786 peptides (Figure 3. A lower panel) was exclusive to CPT-treated cells, and amino acids at position 2 were predominantly Proline (P), Histidine (H), or Arginine (R), while the most amino acids at position-9 were Leucine (L), Tyrosine (Y), or Phenylalanine (F). This was in stark contrast to the predominant occupation of position 2 by Leucine (L), or Tyrosine (Y) and position 9 by Valine (V), Leucine (L), or Glutamic acid (E) in the majority of the peptides associated with MHC I molecules in SW620 cells irrespective of the treatment status.

### Cell component selection for peptide display in control and treated cells shows similarities and differences in the overall pattern and proportion

**Figure 4.**
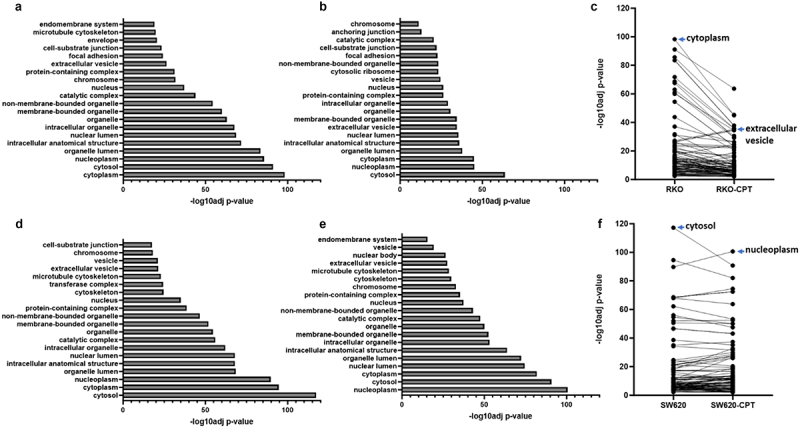
Gene ontology (GO) comparison of proteins displayed from cellular components (CC). Parental proteins for peptides displayed on control or CPT-treated colon cancer cells were categorized based on the gene ontology classification of cellular components. Data for RKO proteins are shown in a–c, as control (a), treated (b) and paired comparison (c) of -log10 adjusted p-values for the top 20 CC categories are shown on X-axis. D–F represent parallel data from SW620 cells, as control (d), treated (e), and paired comparison (f).

MHC I molecules associate with peptides derived from the proteolysis of endogenous proteins. Therefore, we next analyzed the nature and diversity of the peptides associated with MHC I molecules. For this, we sorted the parental proteins from which the peptides were derived, and used gene ontology (GO) classification criteria to determine the cellular components sampled for proteolytic processing and MHC presentation in either control or CPT-treated cells. As shown in Figure 4, the overall sampling diversity of cellular components showed a marked decrease in CPT treated RKO cells (panels **A-C**) while it remained stable in SW620 cells (**panels D-F**). Here, drop in *p*-value for a given GO category correlates with lower number of protein species identified in the particular group. As a result, while the overall diversity of sampling fell or remained stable, a small number of GO categories still showed increased number of proteins sampled and associated with MHC I molecules. These included proteins in the GO categories of extracellular vesicles in RKO cells and nucleus-associated cellular components in SW620 cells (Figure 4c,f). On the other hand, there was a marked drop in the sampling of cytoplasmic and cytosolic proteins in both cells.

### RKO and SW620 cells process overlapping as well as distinct proteins for display on MHC I

Next, we analyzed if the two cell types processed same types of proteins for association with MHC I and display on cell surface. For this analysis, we chose from each cell type only those top 10 proteins from which 6 or more peptides were generated by proteolysis and were detected in our study.

As a result, the most number of peptides per protein were generated in RKO cells, in which HS71A and HS90B proteins generated 19 distinct peptides each, with 14 of the 19 hS71A peptides and 9 of the 19 hS90B peptides being generated only in CPT-treated cells (Table 3). Also noted was that 1–4 peptides from each of HS71A, HS90B, TBA1C, and ENOA proteins were associated with MHC I molecules of both RKO and SW620 cells. Seven of the top 10 RKO proteins were also proteolytically processed by SW620 cells to generate MHC I-associated peptides, but with sequences distinct from those generated by RKO cells. Additionally, the molecular sizes of top 10 proteins processed by RKO cells ranged between 26.7 kDa (RS3 protein) and 102 kDa (TCAF1). Nine of the ten proteins were smaller than 100 kDa in molecular size.

**Table 3.** Top 10 RKO cell proteins with 6 or more peptides displayed on MHC I. Also shown are total number of peptides from each protein, number unique to treated cells, and number of peptides cross-presented on SW620 cells, and if unique peptide from same protein is displayed by SW620.

Cell line	Protein (Accession #)	Mol. Mass (kDa)	# of peptides identified	# of Rx-restricted peptides	# of Cross-presented peptides	Unique peptide from Protein is presented by other cell line
RKO	HS71A (P0DMV8)	70	19	14	2	yes
	HS90B (P08238)	84	19	9	4	yes
	TBA1C (Q9BQE3)	50	10	8	1	yes
	ENOA (P06733)	47	9	4	1	no
	EF2 (P13639)	95.3	8	6	0	yes
	CH60 (P10809)	61	8	5	0	no
	RS3 (P23396)	26.7	8	5	0	no
	TCAF1 (Q9Y4C2)	102	8	3	0	yes
	VIME (P08670)	53.6	7	7	0	yes
	TBB4b (P68371)	49.8	7	4	0	yes

In contrast, the top 10 proteins processed for MHC I presentation by SW620 cells ranged in molecular size between 10.8 kDa (S10A8) and 532 kDa (PLEC), with six of the 10 proteins larger than 120 kDa (Table 4), suggesting a marked difference between the two cells in the preferential sampling of endogenous proteins of variable sizes. The majority of the peptides from the top 10 proteins from SW620 cells were generated only after treatment with CPT. For example, all eight peptides generated from the desmosomal protein Desmoplakin (DESP) and all six peptides from the S100 family protein S10A8 (aka S100A8) were generated in CPT treated cells. Interestingly, in contrast to peptides derived from the top four proteins from RKO cells, none of the peptides from the top 10 proteins in SW620 cells was displayed in RKO cells. However, seven of the 10 proteins from SW620 were also processed in RKO cells for MHC I association, although the peptides sequences were distinct from those displayed in SW620 cells.

**Table 4.** Top 10 SW620 cell proteins with six or more peptides displayed on MHC I. Also shown are total number of peptides from each protein, number unique to treated cells, and if unique peptide from same protein is displayed by RKO cells. None of the peptides identified from top 10 SW620 proteins was similarly displayed by RKO cells.

Cell line	Protein (Accession #)	Mol. Mass (kDa)	# of peptides identified	# of Rx-restricted peptides	# of Cross-presented peptides	Unique peptide from Protein is presented by other cell line
SW620	PRP8 (Q6P2Q9)	230–280	15	9	0	yes
	DDB1 (Q16531)	127	11	7	0	yes
	S10A9 (P06702)	13.2	10	9	0	no
	PLEC (Q15149)	532	8	4	0	yes
	DESP (P15924)	250–330	8	8	0	yes
	NONO (Q15233)	54	7	5	0	yes
	MYO1B (O43795)	132	6	5	0	yes
	S10A8 (P05109)	10.8	6	6	0	no
	CDK1 (P06493)	36.2	6	5	0	no
	IQGA1 (P46940)	189	6	5	0	yes

### Peptides displayed on MHC I may be surface accessible on the parent protein

**Figure 5.**
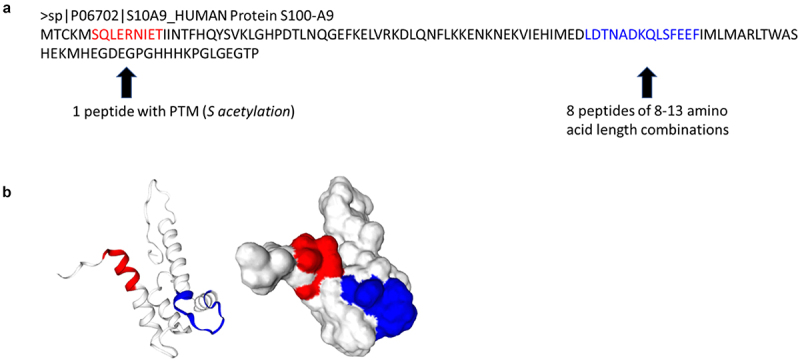
Sequence and topography of S100-A9 protein with depiction of peptides displayed on MHC I of treated SW620. (a) mapping of human S100-A9 peptide sequences. The human protein S100-A9 sequence is shown. Left arrow points to a peptide sequence in red that was displayed on MHC I and has a post-translational modification (PTM) identified as acetylation. The arrow on the right points to the sequence shown in blue, that was the origin of eight peptides ranging in length from 8 to 13 amino acids that were displayed on MHC I. (b) Topographic representation of the S100-A9 protein, with the peptides displayed on MHC I molecule. A topographic representation of the S100-A9 protein is depicted, showing the three-dimensional structure and the unique peptides (shown in color) that were presented on MHC I molecules in treated SW620 cells. The peptides appear to be surface accessible for external molecular interactions.

MHC-associated peptides are made of linear amino acids generated by proteolysis of the parent protein. Biochemical structures for a number of proteins or fragments of large size proteins have been resolved and publicly accessible (www.rcsb.org). The availability of these resources offers an opportunity to map peptide three-dimensional location and orientation within the parent protein. As an example of this possibility, we used to map the location of two regions on the protein S10A9 (S100A9) from where nine out of ten peptides were generated in treated SW620 cells. This model generated from the actual structural data showed that the S-acetylated peptide SQLERNIET was derived from an N-terminal alpha helix region of S10A9 protein while the remaining eight MHC I-associated peptides from the same protein were derived from the sequence LDTNADKQLSFEEFI located in an unstructured region between the N-terminal two alpha helices (**Figure 5a**). Although unstructured regions on proteins are harder to solve or predict the structure compared to helices or sheets, 3D topography of these peptide locations suggests at least partial surface-accessibility of these peptides on the protein 3D structure (**Figure 5b**). These mapping results indicate that peptides displayed in association with MHC I molecules may be derived from protein regions accessible for interaction with other molecules.

## Discussion

In this report, we identify a repertoire of endogenous peptides displayed in association with MHC I molecules of two colon cancer cell lines of distinct microsatellite stability status and MHC alleles. Through comparison of both the peptides and the parental proteins from which the peptides were derived, we identify distinct as well as shared features involved in the process of peptides displayed in association with MHC I. Additionally, we show that chemotherapy of colon cancer cells may alter the quantitative and qualitative selection, processing, and display of peptides that associate with the MHC I and, therefore, are displayed on the cell surface. Since MHC I molecules are the basis for self-recognition and non-self distinction by T-cells, the identification of therapy-induced distinct molecules marks a step toward studies to identify biomarkers for various states in cancer biology.^34–38^

The selection of peptides to be displayed on the surface of cells requires cytosolic proteolysis of proteins, followed by processing and generation of peptides of specific length and composition, and association of the generated peptides with MHC I molecules in transit to the cell surface. Among others, the MHC I allele, intracellular abundance of the target protein, molecular size of the target protein, the presence of suitable amino acid sequences^39–41^ are factors that determine the type and sequence of peptides displayed in association with MHC I. Any previously unknown MHC-I – peptide complex, a neo-epitope or neo-antigen, could then be visible as non-self, and may activate immune cells that will eliminate specific cells that display the altered MHC-peptide complex. Therefore, strategies to increase neo-antigens and the visibility of cancer cells to immune system have the potential to synergize with immunotherapy, and empower the patient’s own immune system to attack cancer cells.^42–44^

We examined in vitro colon cancer cells treated with a chemotherapy agent, to identify peptides displayed on MHC I, and that are distinct from those processed and displayed by untreated cells. Given the ineffectiveness of the current immunotherapy in CRC and some other solid tumors, we asked if chemotherapy could trigger the display of neo-peptides on cancer cells. In principle, such neo-peptides could initiate the expansion of specific cytotoxic T-cells that may recognize the ‘altered’ cells and target them for elimination. Previous reports have shown that chemotherapy and radiotherapy increase the expression of MHC I molecules on cancer cells.^45–48^ Since increased expression of MHC I on the surface will be accompanied by increased display of endogenous peptides, we were interested in studying the presence and the nature of such peptides.

We anticipated that cells with variable microsatellite and MHC I allele status would display a widely diverse set of peptides. Microsatellite instability (MSI) in CRC, characterized inability to efficiently repair DNA mismatch, enables the persistence of altered DNA sequence and therefore generation of ‘altered self’ endogenous molecules that can be targeted by the immune system. Not surprisingly, immunotherapy has been approved for MSI classified CRC. In contrast, microsatellite stable (MSS) CRC is not amenable to immunotherapy, the mechanisms of which are not fully known. Lack of neo-epitopes/neo-antigens has been proposed as one of the characteristics of MSS tumors.^4–8^

In this immunopeptidome preliminary study, we show that although diverse peptides were displayed by the two CRC cell types, there are common sets of proteins sampled, and common peptides displayed by the two different cell types, albeit a small number relative to the total number of peptides in the pool. Both cell types also displayed peptides generated only after treatment with camptothecin, suggesting that clinical chemotherapy drugs have the potential to enhance the diversity of peptides on MHC and visibility of both MSS and MSI CRC. However, we also noted that although the overall number of peptides increased, the overall sampling frequency within a specific Gene Ontology category decreased, suggesting enhanced processing but not protein sampling for proteolysis.

As mentioned above, the protein molecular size and abundance play role in the intracellular sampling of proteins for proteolysis and MHC-association. In our data, peptides from both small size as well as large-sized proteins were detected, although most of the top 10 proteins sampled in SW620 were large-size proteins. The mechanisms for such differential sampling are intriguing and need to be further investigated. Also, notable is the increased relative sampling of nuclear and nucleoplasmic cellular compartments in CPT-treated MSS SW620 cell line, which raises questions whether DNA-damaging drugs could induce the generation of treatment-restricted peptides preferentially from nuclear proteins. Indeed, a number of the peptides presented in Tables 1 and 2 originated from nuclear proteins.

The possibility that some peptides identified in this study may be accessible on the surface of the parent protein raises the question if the parent protein may be targetable by antibodies, aptamers, or compounds, especially if the protein is membrane- or vesicle-associated. Such peptides and proteins may be developed into biomarker tools that identify cells expressing the protein, or vaccine or research tools to activate immune cells.^36,46^

As a preliminary *in vitro* study, our report has some limitations. First, we do not know if untreated or chemotherapy treated normal cells process and display a similar set of peptides in association with MHC I. In this scenario, cancer cells will not be differentially visible to immune cells, or they may induce autoimmunity. Therefore, development of strategies to narrowly target therapeutic drugs to tumors or to treat autologous cancer cells ex vivo may be necessary to direct the immune system toward cancer cells. Second, the immunogenicity or the potential biomarker utility of any of the peptides generated by treated cells is entirely unknown and therefore needs further study. Third, given the diversity and complexity of MHC-alleles, it is very challenging to study the peptide repertoire for all known alleles. Despite the complexity of both the alleles and the process peptide selection, some peptides may be presented across multiple related alleles and similar protein sampling mechanisms may exist among different cells. Therefore, it is possible that some endogenous peptides and proteins are processed and presented similarly by different cells. Besides, such a complexity may be minimized by using patient autologous cancer cells for intervention strategy. For example, based on the MHC I-profile of the patient, either allele-specific peptides or the parental proteins may be used to guide immune cells to the cancer cells.

Despite these limitations, the identification of MHC-I associated peptides, specifically on colon cancer cells treated with a chemotherapy agent, opens venues for further research to develop potential biomarkers for the selection of patients for immunotherapy, to expand personalized precision medicine by autogenous vaccines or orthotopic induction of immunity, and to develop therapeutic molecules that enhance chemotherapy or immunotherapy.

## Data Availability

The data that support the findings of this study are available from the corresponding author, [TS], upon reasonable request.
